# The combined effects of extended feeding with a high level of *Arthrospira platensis* and a commercial enzyme mix or porcine pancreatin on broilers’ blood cells, plasma metabolites and liver lipid profile

**DOI:** 10.1186/s12917-025-04532-2

**Published:** 2025-07-17

**Authors:** Paula A. Lopes, Cristina M. Alfaia, Maria P. Spínola, Rui M. A. Pinto, José M. Pestana, Mónica M. Costa, J. C. Tavares, Miguel P. Mourato, Beatriz Tavares, Daniela F. P. Carvalho, Cátia F. Martins, Madalena Lordelo, José A. M. Prates

**Affiliations:** 1 Faculdade de Medicina Veterinária, CIISA - Centro de Investigação Interdisciplinar em Sanidade Animal, Universidade de Lisboa, Lisboa, 1300-477 Portugal; 2 Laboratório Associado para Ciência Animal e Veterinária (AL4AnimalS), Lisboa, Portugal; 3 Laboratório de Análises Clínicas Dr. Joaquim Chaves, JCS, Avenida General Norton de Matos, Miraflores, Algés, 1495-148 Portugal; 4 iMED.UL, Faculdade de Farmácia, Universidade de Lisboa, Avenida Professor Gama Pinto, Lisboa, 1649-003 Portugal; 5 Instituto Superior de Agronomia, Universidade de Lisboa, Tapada da Ajuda, Lisboa, 1349-017 Portugal; 6 Environment, Agriculture and Food, Instituto Superior de Agronomia, Associated Laboratory TERRA, LEAF - Linking Landscape, Universidade de Lisboa, Tapada da Ajuda, Lisboa, 1349-017 Portugal; 7 Faculdade de Medicina Veterinária, Avenida da Universidade Técnica, Pólo Universitário do Alto da Ajuda, Lisboa, 1300-477 Portugal

**Keywords:** *Arthrospira platensis*, Long-term feeding, Peptidases, Plasma metabolites, Hepatic lipids, Broilers

## Abstract

**Background:**

The increasing global demand for sustainable and eco-friendly protein alternatives in poultry production has led to the exploitation of unconventional feed ingredients, such as microalgae. This study is novel for its exploration of the extended feeding effects (from day 7 to day 35) of a high inclusion level (15%) of *Arthrospira platensis* (Spirulina) in broiler chickens’ diet, either alone or supplemented with enzymes, primarily peptidases (EC 3.4). The study assessed the impact on the blood cells, metabolic status and the hepatic content of lipids, pigments and minerals. One hundred and twenty Ross 308 male chickens were distributed into 40 battery brooders, housing three birds per cage. Initially, the chickens were given *ad libitum* access to a standard corn and soybean meal-based diet for the first seven days. Subsequently, from day 7 to 35, they were assigned to one of four experimental diets: a control diet based on corn and soybean (control group, *n* = 10), a diet incorporating 15% *A*. *platensis* (SP group, *n* = 10), a diet incorporating 15% *A. platensis* supplemented with 0.025% of the commercial enzyme mix VemoZyme^®^ P (SPV group, *n* = 10), and a diet incorporating 15% *A. platensis* supplemented with 0.10% of porcine pancreatin (SPP group, *n* = 10).

**Results:**

The 15% inclusion of *A. platensis* negatively impacted the birds’ growth performance by decreasing the final body weight (*p* < 0.001), body weight gain (*p* < 0.001) and average daily feed intake (*p* < 0.001), while increasing the feed conversion ratio (*p* = 0.001). This high level of *A. platensis* incorporation did not change the haematological profile but raised blood lipid levels. However, these increases were successfully normalized by supplementing the diet with the enzyme mix VemoZyme^®^ P and the porcine pancreatin. Spirulina positively altered the fatty acid composition in the liver, notably increasing *n-3* PUFA content (*p* < 0.001) and reducing the *n-6*/*n-3* PUFA ratio (*p* < 0.001). Furthermore, including A. platensis augmented the concentration of beneficial pigments with antioxidant functions, irrespective of enzyme addition. Most mineral levels remained unaffected (*p* > 0.05) by *A. platensis*.

**Conclusions:**

Overall, data suggest that the impact of *A. platensis* on blood and liver measurements outweighs the effect of enzyme supplements, VemoZyme^®^ P or porcine pancreatin. However, these enzyme mixtures effectively mitigated the elevated blood lipid levels induced by Spirulina. Although our findings illustrate the potential of *A. platensis* as an alternative protein source of nutrition for poultry, further exploration is necessary to determine the feasibility of higher incorporation levels over the long term, particularly in light of the negative effects on broilers’ growth performance.

## Background

The common feed ingredients, such as soybean meal and cereal grains, are used in poultry diets as the main protein and energy sources, respectively. The availability of these traditional feedstuffs will eventually become both environmentally and economically unsustainable in the near future, due to land use with consequent increases in greenhouse gas emissions, water deprivation, and global climate changes during the subsequent processes of production, harvesting, and storage. The increasing global demand for sustainable and eco-friendly protein alternatives in poultry farming has led to the exploration of unconventional feed ingredients, with microalgae, particularly those from the Spirulina genus, garnering significant attention [1–5]. *Arthrospira platensis*, commonly known as Spirulina, is a cyanobacterium valued for its high-quality protein, carbohydrates, essential fatty acids, vitamins, pigments and minerals. However, these contents are subject to variation based on cultivation requirements and seasonal changes [6, 7].

Beyond its robust nutritional profile, *A. platensis* also exhibits functional benefits including antimicrobial, antioxidant, anti-inflammatory and immunomodulatory properties [7–9]. Despite these benefits, the microalga’s largely indigestible cell wall, composed mostly of peptidoglycan and lipopolysaccharides akin to Gram-negative bacteria, poses challenges to monogastric animals [10]. Furthermore, the digestibility of algal proteins can be inhibited due to protein-pigment complexes, such as phycocyanins attached to thylakoid membranes [11].

Although carbohydrases, like lysozyme, can partially disrupt the *A. platensis* cell wall [12], a gelation process seems to encase nutrients, inhibiting their digestion [3, 13]. While some endeavours have sought to hydrolyse *A. platensis* proteins through physical pre-treatments paired with pancreatin or trypsin [14, 15], using such enzymes to degrade proteins in *A. platensis* feed for monogastric animals remains uncharted territory. Nonetheless, studies have shown the potential effectiveness of specific exogenous enzymes, such as carbohydrate-degrading ones, in disrupting the *A. platensis* cell wall and enhancing the bioavailability of beneficial nutritional compounds [16]. The use of these exogenous enzymes as feed additives in poultry diets is a common strategy to overcome the lack of endogenous enzymes and negate anti-nutritional factors, thereby improving the nutrient digestibility of dietary components [17, 18]. Such enzymes also generate prebiotic oligosaccharides, promoting gut health and growth parameters [19].

Historically, most studies have incorporated microalgae as a supplement, rather than an ingredient, in monogastric diets [20]. Our previous research evaluated the impact of integrating microalgae as a feed ingredient on broilers’ growth performance, health status, and hepatic lipid composition [4, 5], as well as on pigs [21, 22]. This study breaks new ground by examining the combination of long-term feeding with a high level of *A. platensis*, individually or supplemented with different peptidases (EC 3.4), on broilers’ health and hepatic lipid metabolism.

Our goal was to assess the impact of replacing 15% of soybean meal with *A. platensis*, used alone or in conjunction with the commercial enzyme mix VemoZyme^®^ P or porcine pancreatin, from day 7 to day 35, on broilers’ blood cells, plasma metabolites and hepatic composition, encompassing lipids, antioxidant diterpenes, pigments and mineral profile. In this study, we hypothesised that a high level of dietary incorporation of *A. platensis* during an extended feeding period supplemented with the enzyme mixes mentioned above improves the systemic and hepatic lipid metabolism in broilers.

## Results

### Effect of experimental diets on birds’ growth performance and feed intake

**Fig. 1:**
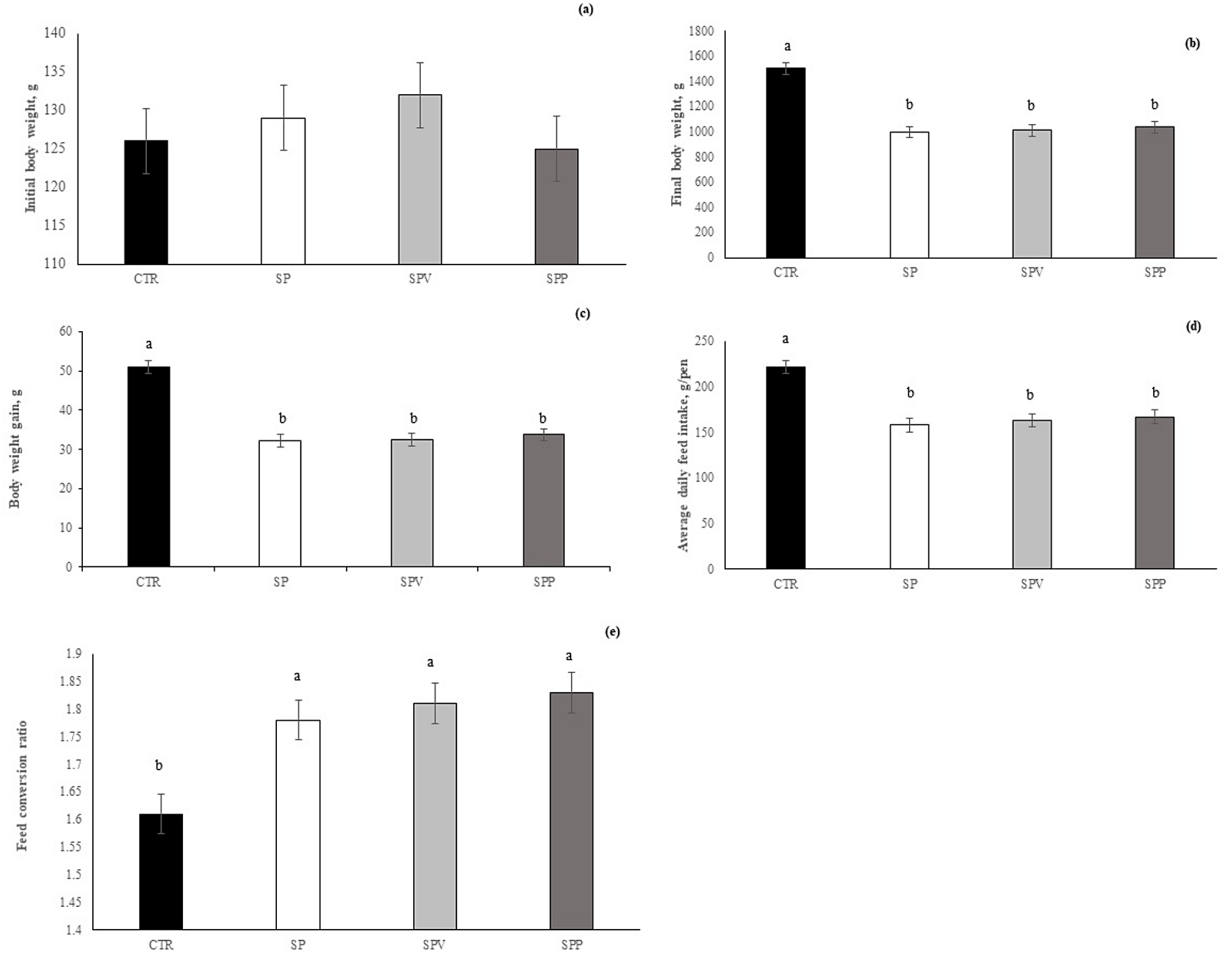
Influence of experimental diets on growth performance parameters of broilers. **(a)** initial body weight (g); **(b)** final body weight (g); **(c)** body weight gain (g); **(d)** average daily feed intake (g); **(e)** feed conversion ratio. Diets: Control (corn/soybean), SP (15% *A. platensis*), SPV (15% *A. platensis* plus 0.025% VemoZyme^®^ P), SPP (15% *A. platensis* plus 0.10% porcine pancreatin). Values are presented as mean and SEM (standard error of the mean). ^a.b^Different superscripts indicate a significant difference (*p* < 0.05)

Data on birds’ growth performance and feed intake are shown in Fig. 1. Final body weight was reduced by the addition of *A. platensis*, with or without the enzyme supplementation (*p* < 0.001), relative to control birds. The same variation was observed for body weight gain (*p* < 0.001) and average daily feed intake (ADFI) (*p* < 0.001). Conversely, the feed conversion ratio was increased in *A. platensis* diets regardless of the control diet’s enzyme supplementation (*p* = 0.001).

### Effect of experimental diets on blood cells count and plasma metabolites

Blood cell count and plasma biochemical profile of broilers fed 15% of *A. platensis*, supplemented or not with the VemoZyme^®^ P and porcine pancreatin, are displayed in Table 1. The counts for red blood cells (*p* = 0.610), haemoglobin (*p* = 0.337) and thrombocytes (*p* = 0.305) were not affected by dietary changes. Although the total count of white blood cells didn’t show significant variation (*p* = 0.102) with the addition of Spirulina, a noticeable increase in the percentage of granulocytes (*p* = 0.014) was observed in the presence of Spirulina, whether alone or in combination with VemoZyme^Ⓡ^ P, relative to the control. This was contrasted by a decrease in lymphocyte percentages (*p* = 0.022). A decrease in the percentage of monocytes (*p* = 0.045) was also recorded when Spirulina was combined with VemoZyme^Ⓡ^ P compared to the control.

**Table 1:** Blood count and plasma metabolites of broilers fed the experimental diets

	Control	SP	SPV	SPP	SEM	*p*-value
White blood cells (x10^9^/L)	14.7	5.26	6.07	13.3	3.48	0.102
Granulocytes (%)	34.0^b^	47.6^a^	48.9^a^	43.5^a, b^	3.35	0.014
Lymphocytes (%)	63.0^a^	50.7^b^	50.0^b^	55.3^a, b^	3.18	0.022
Monocytes (%)	3.00^a^	1.71^a, b^	1.14^b^	1.17^a, b^	0.508	0.045
Red blood cells (x10^12^/L)	2.88	3.25	3.14	3.48	0.334	0.610
Haemoglobin (g/dL)	9.22	9.94	9.76	10.1	0.364	0.337
Thrombocytes (x10^9^/L)	26.2	22.7	22.7	20.5	2.15	0.305
Glucose (mg/dL)	223^b^	244^ab^	255^a^	247^a^	5.88	0.004
Urea (mg/dL)	3.27^a^	1.95^b^	1.77^b^	2.16^b^	0.189	< 0.001
Creatinine (mg/dL)	0.012	0.017	0.011	0.016	0.002	0.054
Cholesterol (mg/dL)	110^b^	140^a^	107^b^	99.5^b^	3.82	< 0.001
LDL-CHR (mg/dL)	18.4^b^	30.7^a^	15.9^b^	19.3^b^	1.27	< 0.001
HDL-CHR (mg/dL)	75.3^b^	83.5^a^	70.7^b^	67.9^b^	2.15	< 0.001
VLDL-CHR (mg/dL)	10.0^b^	14.4^a^	13.2^a^	10.0^b^	0.609	< 0.001
Triacylglycerols (mg/dL)	50.2^b^	72.0^a^	65.8^a^	50.1^b^	3.04	< 0.001
Total lipids (mg/dL)	420^b^	501^a^	430^b^	399^b^	8.81	< 0.001
ALT (U/L)	1.20	1.30	1.10	1.00	0.113	0.288
AST (U/L)	276^a^	166^b^	148^bc^	144^c^	4.97	< 0.001
GGT (U/L)	19.9^b^	24.3^a^	20.0^b^	18.2^b^	0.814	< 0.001
ALP (U/L)	1501^c^	1696^a^	1506^c^	1600^b^	19.8	< 0.001
Total protein (g/dL)	2.49	2.46	2.53	2.49	0.025	0.216
C-reactive protein (mg/dL)	0.009^ab^	0.012^a^	0.005^b^	0.005^b^	0.001	< 0.001
Na^+^ (mEq/L)	147^ab^	150^a^	147^ab^	145^b^	1.26	0.026
K^+^ (mEq/L)	6.62	6.25	6.69	6.83	0.373	0.725
Cl^−^ (mEq/L)	108	111	110	107	1.18	0.142

Diets: Control (corn/soybean), SP (15% *A. platensis*), SPV (15% *A. platensis* plus 0.025% VemoZyme^®^ P), SPP (15% *A. platensis* plus 0.10% porcine pancreatin)

TAG - triacylglycerols; HDL - high-density lipoproteins; LDL - low-density lipoproteins; VLDL - very low-density lipoproteins; ALT - alanine aminotransferase (EC 2.6.1.2); AST - aspartate aminotransferase (EC. 2.6.1.1); ALP - alkaline phosphatase (EC 3.1.3.1); GGT - gamma-glutamyltransferase (EC 2.3.2.13)

Total lipids = [total cholesterol] × 1.12 + [TAG] × 1.33 + 148, as described by Covaci et al. [62]

VLDL-CHR = 1/5 [TAG], as described by Friedewald et al. [61]

All values are presented as the mean and SEM (standard error of the mean).^a.b, c^Different superscripts within a row indicate a significant difference (*p* < 0.05)

In the context of plasma metabolites, glucose levels peaked in broilers fed Spirulina with VemoZyme^®^ P compared to the control group (*p* = 0.004), while urea displayed the opposite change (*p* < 0.001). No variations were observed among experimental treatments for creatinine (*p* = 0.054).

The study further showed that broilers fed exclusively with 15% Spirulina had elevated levels of total cholesterol (*p* < 0.001), LDL-cholesterol (*p* < 0.001), HDL-cholesterol (*p* < 0.001), VLDL-cholesterol (*p* < 0.001), triacylglycerols (*p* < 0.001) and total lipids (*p* < 0.001), in comparison to the other experimental groups.

In terms of liver function, ALT levels were comparable across experimental groups (*p* = 0.288). However, AST levels were found to be elevated in broilers on the control diet (*p* < 0.001). The enzymes ALP (*p* < 0.001) and GGT (*p* < 0.001) displayed higher activities in broilers fed exclusively *A. platensis*, compared to control birds and those fed *A. platensis* supplemented with VemoZyme^®^ P or porcine pancreatin. Total protein levels remained unchanged across diets (*p* = 0.216). The acute phase protein, C-reactive protein (*p* = 0.000), was increased by individual Spirulina relative to the other Spirulina diets.

In assessing the major electrolytes, sodium increased in broilers fed only *A. platensis*, relative to broilers fed *A. platensis* plus porcine pancreatin (*p* = 0.026). Conversely, no significant changes were observed for potassium (*p* = 0.725) and chloride (*p* = 0.142).

### Effect of experimental diets on total lipids, cholesterol and fatty acid profile in the liver

The effect of the experimental diets on the total lipids, total cholesterol and fatty acid composition in the liver of broilers are displayed in Table 2. The diets included 15% of *A. platensis* supplementation, with or without the addition of the enzyme mixes VemoZyme^®^ P and porcine pancreatin. Total cholesterol levels did not show significant fluctuations across the dietary treatments (*p* > 0.05). However, a distinct reduction in total lipid levels was observed in broilers fed with 15% of *A. platensis*, either alone or in combination with the VemoZyme^Ⓡ^ P or porcine pancreatin, when compared to the control group (*p* < 0.001).

**Table 2:** Hepatic total lipids, total cholesterol and fatty acid (FA) profile of broilers fed the experimental diets

Item	Control	SP	SPV	SPP	SEM	*p*-value
***Total lipids***,*** g/100 g***	3.23^a^	2.30^b^	2.30^b^	2.34^b^	0.148	< 0.001
***Total cholesterol***,*** mg/g***	2.57	2.20	2.41	2.14	0.137	0.120
***FA composition***,*** g/100 g FA***						
12:0	0.022	0.012	0.015	0.010	0.004	0.117
14:0	0.287^a^	0.168^b^	0.166^b^	0.168^b^	0.027	0.005
14:1*c*9	0.019	0.008	0.008	0.011	0.006	0.568
15:0	0.044	0.042	0.045	0.051	0.004	0.483
16:0	18.4	18.7	19.1	18.9	0.625	0.884
16:1*c*7	0.509	0.478	0.418	0.445	0.040	0.414
16:1*c*9	0.529	0.962	0.957	0.979	0.151	0.119
17:0	0.246	0.272	0.305	0.309	0.022	0.167
17:1*c*9	0.023^b^	0.038^a^	0.038^a^	0.036^ab^	0.003	0.013
18:0	23.5	21.9	22.1	21.9	0.611	0.194
18:1*c*9	20.2	16.1	15.5	15.4	1.63	0.142
18:1*c*11	1.07^b^	1.64^a^	1.55^a^	1.60^a^	0.071	< 0.001
18:2*n-6*	17.7	15.4	15.8	15.6	0.680	0.086
18:3*n-6*	0.046	0.033	0.035	0.036	0.004	0.097
18:3*n-3*	0.069^b^	0.127^a^	0.133^a^	0.130^a^	0.010	< 0.001
20:0	0.091	0.106	0.093	0.088	0.006	0.210
20:1*c*11	0.251	0.305	0.303	0.296	0.018	0.128
20:2*n-6*	0.676	0.477	0.529	0.566	0.057	0.078
20:3*n-6*	1.55^b^	2.39^a^	2.24^a^	2.26^a^	0.139	0.001
20:4*n-6*	9.96^b^	14.6^a^	14.2^a^	14.5^a^	1.05	0.008
20:3*n-3*	0.017^b^	0.029^a^	0.031^ab^	0.031^ab^	0.003	0.003
20:5*n-3*	0.037^b^	0.253^a^	0.190^a^	0.193^a^	0.019	< 0.001
22:0	0.046	0.066	0.060	0.063	0.006	0.103
22:1*n*-9	0.017	0.027	0.026	0.034	0.004	0.060
23:0	0.062^b^	0.094^a^	0.096^a^	0.091^a^	0.006	0.001
22:5*n-3*	0.202^b^	0.569^a^	0.619^a^	0.619^a^	0.051	< 0.001
22:6*n-3*	0.470^b^	0.971^a^	1.17^a^	1.08^a^	0.096	< 0.001
Others	3.94	4.30	4.29	4.50	0.384	0.778
Σ SFA^1^	42.8	41.3	41.9	41.7	0.619	0.425
Σ MUFA^2^	22.6	19.6	18.8	18.8	1.81	0.410
Σ PUFA^3^	30.7	34.8	34.9	35.0	1.60	0.176
Σ *n-6* PUFA^4^	29.9	32.8	32.8	33.0	1.49	0.405
Σ *n-3* PUFA^5^	0.797^b^	1.95^a^	2.14^a^	2.06^a^	0.141	< 0.001
PUFA/SFA	0.725	0.845	0.833	0.839	0.041	0.145
*n-6*/*n-3*	40.8^a^	17.0^b^	15.8^b^	16.6^b^	1.71	< 0.001

Diets: Control (corn/soybean); SP (15% *A. platensis*); SPV (15% *A. platensis* plus 0.025% VemoZyme^®^ P); SPP (15% *A. platensis* plus 0.10% porcine pancreatin)

^1^SFA (saturated fatty acids) - Sum (12:0, 14:0, 15:0, 16:0, 17:0, 18:0, 20:0, 22:0, 23:0);

^2^MUFA (monounsaturated fatty acids) - Sum (14:1*c*9, 16:1*c*7, 16:1*c*9, 17:1*c*9, 18:1*c*9, 18:1*c*11, C0:1*c*11, 22:1*n*-9);

^3^PUFA (polyunsaturated fatty acids) - Sum (18:2*n-6*, 18:3*n-6*, 18:3*n-3*, 20:2*n-6*, 20:3*n-6*, 20:4*n-6*, 20:3*n-3*, 20:5*n-3*, 22:5*n-3*, 22:6*n-3*);

^4^*n-6* PUFA - Sum (18:2*n-6*, 18:3*n-6*, 20:2*n-6*, 20:3*n-6*, 20:4*n-6*);

^5^*n-3* PUFA - Sum (18:3*n-3*, 20:3*n-3*, 20:5*n-3*, 22:5*n-3*, 22:6*n-3*)

All values are presented as the mean and SEM (standard error of the mean)

^a, b^Different superscripts within a row indicate a significant difference (*p* < 0.05)

Dietary interventions also substantially affected the fatty acid profile. The major fatty acids, listed in descending order of presence, were 18:0, 18:1*c*9, 16:0, 18:2*n-6* and 20:4*n-6*. Notably, broilers that consumed 15% *A. platensis*, with or without the supplemental enzymes, exhibited lower amounts of 14:0 compared to the control (*p* = 0.005). Concurrently, consumption of *A. platensis* resulted in a significant increase in the presence of several other fatty acids, namely 18:1*c*11, 18:3*n-3*, 20:3*n-6*, 20:4*n-6*, 20:5*n-3*, 22:5*n-3* and 22:6*n-3* (*p* < 0.001), irrespective of the enzymes addition. The highest values of 17:1*c*9 (*p* = 0.013) were found in broilers fed with *A. platensis*, either alone or combined with VemoZyme^®^ P. *A. platensis*-only diet led to an increased amount of 20:3*n-3* (*p* = 0.003) in broilers relative to the control group.

Evaluation of the partial sum of fatty acid percentages revealed that SFA, MUFA, PUFA and *n-6* PUFA levels were not altered by the experimental diets (*p* > 0.05). However, a remarkable increase in *n-3* PUFA was recorded (*p* < 0.001) for broilers fed with *A. platensis*, with or without enzyme supplementation, when compared to the control. The *n-6*/*n-3* PUFA ratio displayed an inverse relationship (*p* < 0.001), whereas the PUFA/SFA ratio remained statistically unaltered across different dietary interventions (*p* = 0.145).

### Effect of experimental diets on vitamin E compounds and pigments in the liver

The diterpene profile and pigments in the liver of broilers fed 15% *A. platensis*, supplemented or not with VemoZyme^®^ P and porcine pancreatin, are shown in Table 3. No significant changes were observed in the levels of tocopherol compounds across different diets (*p* > 0.05). However, the quantities of β-carotene and chlorophyll-*a* saw a significant surge in broilers who consumed *A. platensis*, irrespective of enzyme supplementation (*p* < 0.001). There were no notable variations in the levels of chlorophyll-*b* (*p* = 0.052).

**Table 3:** Hepatic vitamin E homologues and pigments of broilers fed the experimental diets

Item	Control	SP	SPV	SPP	SEM	*p*-value	
**Diterpene profile, µg/g**							
α-Tocopherol	7.64	8.20	8.71	7.38	0.550	0.338	
γ-Tocopherol + β-Tocotrienol	0.256	0.222	0.234	0.214	0.0141	0.170	
***Pigments***,*** µg/100 g***							
β-Carotene	50.8^b^	2990^a^	3045^a^	3017^a^	5.40	< 0.001	
Chlorophyll-*a*^1^	16.5^b^	38.8^a^	47.5^a^	33.8^a^	4.13	< 0.001	
Chlorophyll-*b*^2^	28.6	39.6	37.0	27.6	3.46	0.052	
Total chlorophylls^3^	45.1^b^	78.4^a^	84.5^a^	61.4^ab^	7.14	0.002	
Total carotenoids^4^	207^c^	1781^b^	2191^a^	1856^b^	80.9	< 0.001	
Total chlorophylls + carotenoids^5^	252^c^	1859^b^	2275^a^	1917^b^	82.6	< 0.001	

Diets: Control (corn/soybean); SP (15% *A. platensis*); SPV (15% *A. platensis* plus 0.025% VemoZyme^®^ P); SPP (15% *A. platensis* plus 0.10% porcine pancreatin)

All values are presented as the mean and SEM (standard error of the mean)

^1^Ca = 11.24 × A_662_ − 2.04 × A_645_

^2^Cb = 0.13 × A_645_ − 4.19 × A_662_

^3^Ca+b = 7.05 × A_662_ + 18.09 × A_645_

^4^Cx+c = (1000 × A_470_ − 1.90 × Ca − 63.14 × Cb) / 214

^5^(Ca + b) + (Cx + c)

^a, b^Means with different superscripts are significantly different (*p* < 0.05)

The total chlorophyll content was significantly higher in broilers fed with *A. platensis*, either solely or in conjunction with VemoZyme^®^ P, compared to the control birds (*p* = 0.002). The pattern of variations for total carotenoids (*p* < 0.001) and the sum of total chlorophylls and carotenoids (*p* < 0.001) was identical across all experimental diets. The highest values were seen in broilers fed Spirulina alongside VemoZyme^Ⓡ^ P. Intermediate values were observed in those who consumed *A. platensis* alone or in combination with porcine pancreatin. Control birds demonstrated the lowest value.

### Effect of experimental diets on macro- and microminerals content in the liver

Table 4 demonstrates the effect of experimental diets on the macro- and micromineral content in the liver of broilers fed *A. platensis*, both with and without the supplementation of VemoZyme^®^ P and porcine pancreatin.

**Table 4:** The hepatic mineral content of broilers fed the experimental diets

Item	Control	SP	SPV	SPP	SEM	*p*-value	
**Macrominerals, mg/100 g**							
Calcium	16.4	16.9	17.2	18.6	0.670	0.125	
Magnesium	14.0^a^	12.0^b^	12.6^ab^	12.4^b^	0.380	0.004	
Potassium	367	354	356	355	5.90	0.417	
Phosphorous	318	326	327	315	6.40	0.473	
Sodium	126^b^	129^ab^	131^ab^	137^a^	2.30	0.018	
Sulphur	209	206	206	205	3.80	0.875	
Total	1050	1044	1050	1042	13.0	0.963	
***Microminerals***,*** mg/100 g***							
Copper	0.416	0.448	0.442	0.438	0.019	0.680	
Iron	23.4	35.2	30.2	33.6	5.18	0.391	
Manganese	0.380	0.383	0.384	0.392	0.018	0.968	
Zinc	2.39	2.31	2.33	2.37	0.061	0.764	
Total	26.5	38.4	33.4	36.8	5.17	0.392	
***Total macro- and microminerals***	1076	1082	1084	1079	14.0	0.982	

Diets: Control (corn/soybean); SP (15% *A. platensis*); SPV (15% *A. platensis* plus 0.025% VemoZyme^®^ P); SPP (15% *A. platensis* plus 0.10% porcine pancreatin)

All values are presented as the mean and SEM (standard error of the mean)

^a, b^Means with different superscripts are significantly different (*p* < 0.05)

Overall, the total macromineral content did not show any significant variation across the experimental diets (*p* = 0.963). Similarly, no significant differences were observed in the levels of calcium (*p* = 0.125), potassium (*p* = 0.417), phosphorus (*p* = 0.473) and sulphur (*p* = 0.875). However, magnesium levels were noticeably lower in birds fed with *A. platensis* alone or in combination with porcine pancreatin, as compared to the control group (*p* = 0.004). The sodium content was found to be elevated in birds consuming Spirulina paired with porcine pancreatin, relative to the control diet birds (*p* = 0.018). As for microminerals, no significant variations were observed for individual microminerals or total microminerals across different diets (*p* > 0.05).

### Principal component analysis

**Fig. 2:**
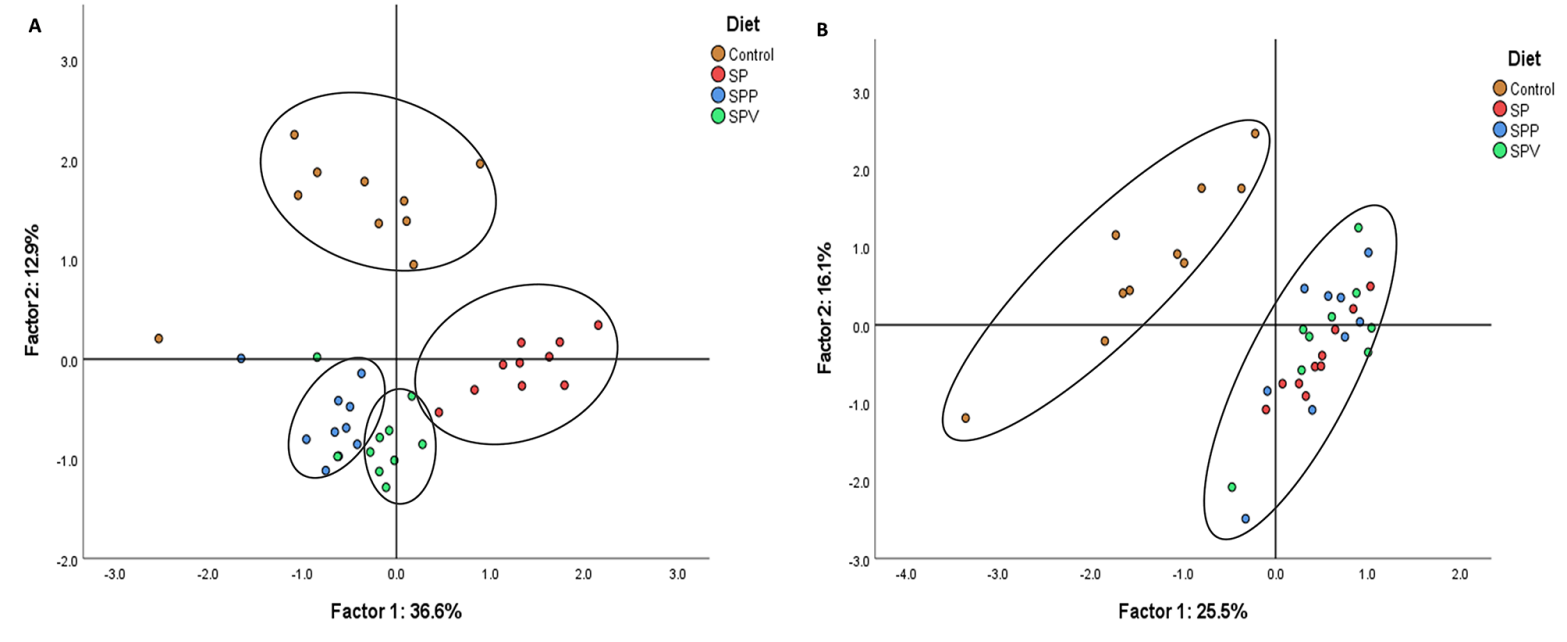
Principal component analysis of plasma biochemical profile (**A**) and liver (**B**) of experimental broilers groups. Diets: Control (corn/soybean), SP (15% *A. platensis*), SPV (15% *A. platensis* plus 0.025% VemoZyme^®^ P), SPP (15% *A. platensis* plus 0.10% porcine pancreatin)

The Principal Component Analysis (PCA) using broilers’ plasma metabolites and hepatic composition reveals distinct variability patterns across the experimental groups (Fig. 2). The score plot in Fig. 2A visualizes the multivariate distribution of the control, SP, SPV and SPP experimental groups based on their plasma metabolites, covering a significant 49.5% of the total data variance (36.6% for Factor 1, and 12.9% for Factor 2). The two factors contribute to a clear segregation of the experimental groups. Table 5 shows the loadings for each variable, with total lipids (0.966), cholesterol (0.917), HDL-cholesterol (0.856), and LDL-cholesterol (0.814) being the most influential variables for Factor 1, and AST (0.868) and urea (0.725) for Factor 2.

**Table 5:** Loadings of the first two principal components of plasma metabolites and liver chemical composition

	Plasma metabolites			Liver	
Variables	Factor 1	Factor 2	Variables	Factor 1	Factor 2
Glucose	0.340	-0.558	Total lipids	-0.847	-0.089
Urea	-0.195	0.725	Cholesterol	-0.089	0.458
Creatinine	0.151	-0.051	12:0	-0.488	0.127
Cholesterol	0.917	0.199	14:0	-0.847	-0.194
LDL	0.824	0.128	14:1*c*9	-0.584	-0.451
HDL	0.856	0.328	15:0	-0.377	-0.610
VLDL	0.760	-0.352	16:0	-0.206	0.049
Total lipids	0.966	0.063	16:1*c*7	-0.037	-0.886
Triacylglycerols	0.760	-0.352	16:1*c*9	0.578	0.414
ALT	0.451	0.273	17:0	0.471	-0.340
AST	-0.144	0.868	18:0	0.026	0.680
GGT	0.798	0.120	18:1*c*9	-0.756	-0.549
ALP	0.534	-0.314	18:1*c*11	0.550	-0.640
Total protein	-0.181	0.025	18:2*n-6*	-0.004	0.693
C-reactive protein	0.569	0.220	18:3*n-6*	-0.052	0.528
Na^+^	0.590	0.245	18:3*n-3*	0.590	-0.293
K^+^	-0.366	-0.102	20:0	0.197	-0.064
Cl^−^	0.279	0.051	20:1*c*11	0.215	-0.464
			20:2*n-6*	0.047	0.767
			20:3*n-6*	0.646	-0.241
			20:4*n-6*	0.838	0.342
			20:3*n-3*	0.699	0.170
			20:5*n-3*	0.544	-0.571
			22:0	0.696	0.261
			22:1*n*-9	0.486	-0.037
			23:0	0.818	0.112
			22:5*n-3*	0.827	0.007
			22:6*n-3*	0.859	0.149
			α-Tocopherol	0.140	0.074
			γ-Tocopherol	-0.086	0.407
			β-Carotene	0.817	-0.480
			Chlorophyll-*a*	0.622	-0.287
			Chlorophyll-*b*	0.335	-0.074
			Total carotenoids	0.795	-0.429
			Sodium	0.282	-0.286
			Potassium	-0.155	0.386
			Calcium	0.167	-0.037
			Magnesium	-0.390	0.561
			Phosphorous	0.173	0.242
			Sulphur	0.007	0.392
			Copper	0.396	0.328
			Zinc	0.005	0.181
			Iron	0.472	0.246
			Manganese	-0.052	0.120

In addition, the score plot in Fig. 2B showcases the location of the experimental groups based on the chemical composition of their livers. Here, the control and SP diets led to the formation of two distinct clusters. The control group was primarily positioned in quadrants *a* and *c*, while the *A. platensis*-based diets, both with and without exogenous enzyme supplementation, were in quadrants *b*, *c* and *d*. In terms of discrimination power, the most impactful variables (Table 5) were 22:6*n-3* (0.859), 20:4*n-6* (0.838), 22:5*n-3* (0.827), 23:0 (0.818) and β-carotene (0.817) for Factor 1, and 16:1*c*7 (0.886), 20:2*n-6* (0.767), 18:2*n-6* (0.693) and 18:0 (0.680) for Factor 2.

## Discussion

The performance of broilers is influenced by several factors, including the level of *A. platensis* incorporated in the diet, the duration of the feeding period and the broilers’ ability to digest *A. platensis* cell walls. In a previous study, Park et al. [2] demonstrated that dietary supplementation of *A. platensis* at low levels (0.25, 0.5, 0.75 or 1.0%) for a period of 35 days resulted in improved feed efficiency in broiler chickens. Conversely, our team observed a decline in the final body weight and body weight gain (BWG), as well as an increase in the feed conversion ratio (FCR), in birds fed with a 15% *A. platensis* diet, both alone and in combination with exogenous enzymes (lysozyme and a mixture of carbohydrate-degrading enzymes) for 21 days [3]. In the current study, dietary supplementation of the same 15% level of *A. platensis*, with or without commercial enzyme mixtures containing peptidases, VemoZyme^®^ P and porcine pancreatin, led to a negative impact on broilers’ growth performance after four weeks of feeding. This was demonstrated by the decreased final body weight, BWG and average daily feed intake (ADFI), and the increased FCR. Becker [23] suggested that poultry diets should limit the inclusion of microalgae to 10% of feed, as higher levels might lead to adverse effects over time, such as increased FCR. This diminished bird performance when higher levels of Spirulina are incorporated could be linked to protein gelation, which potentially results in decreased amino acid digestibility and increased viscosity [13]. Furthermore, the protein content in Spirulina (50–70%) may be resistant to the proteolytic action of birds’ endogenous peptidases [24]. It is worth noting that supplementing the diet with exogenous peptidases did not ameliorate the decline in animal growth performance. *A. platensis*, rich in oligosaccharides, is known to exhibit a prebiotic effect, altering gut fermentation. However, in this study, these effects did not compensate for the negative impact on body weight and ADG, hence the growth impairment was not prevented [25]. This suggests that the animals could not access these intracellular compounds, highlighting the need for a more comprehensive understanding of dietary impacts and nutrient utilization in broilers.

The inclusion of *A. platensis* at a high level in the diet resulted in no significant changes in the majority of blood cell counts, suggesting an unaltered haematological profile. This outcome contradicts the findings of Sugiharto et al. [26], who noted reduced values of haemoglobin, erythrocytes and haematocrit in birds fed a diet containing only 1% of *A. platensis* for 35 days. While the overall white blood cell count remained unaffected across the experimental diets, we observed a distinct variation in granulocyte and lymphocyte counts. Concurrently, the shift in monocyte count mirrored that of the lymphocytes. These fluctuations align with the understanding that white blood cells consist of both granulocytes (neutrophils, eosinophils and basophils) and non-granulocytes (lymphocytes and monocytes). White blood cells are integral to the body’s immune response, and anomalies in their count can indicate the presence of infectious or inflammatory diseases, leukaemia, lymphoma and bone marrow disorders [27]. Interestingly, the addition of *A. platensis* to the diet, either alone or in combination with the VemoZyme^®^ P, led to an increase in granulocyte percentage compared to the control birds. This uptick was immediately offset by a decrease in the percentage of lymphocytes and monocytes in the same experimental groups. This pattern suggests an immune response balance induced by *A. platensis*, corroborating prior studies [26, 28, 29]. Recently, considerable attention has been paid to the immunomodulatory effects of Spirulina when used as a poultry feed additive. Its potential to enhance disease resistance and improve growth rates, especially under stress conditions, underscores its value [29]. The insights gained from this study further validate the importance of exploring the role of dietary components in modulating immune response and overall health outcomes in broilers.

Plasma biochemical metabolites provide invaluable insights into a bird’s overall clinical condition and physical state [30]. In our study, we observed that the inclusion of *A. platensis* in the diet led to an increase in glucose levels in birds that were fed both enzymes. This likely correlates with the known carbohydrate-rich composition of *A. platensis* [22, 31]. Interestingly, while creatinine levels remained unaffected across all experimental diets, a significant reduction in urea was noted in birds fed *A. platensis*, irrespective of supplementation with VemoZyme^®^ P or porcine pancreatin. This could imply that the addition of *A. platensis* did not adversely affect renal function, suggesting a potential benefit of Spirulina in preserving kidney health in broilers. In addition, the decreased urea in birds fed with *A. platensis* could result from its lower protein digestibility.

Regarding the hepatic parameters, except ALT, which exhibited no variations across experimental diets, the changes observed in AST, ALP and GGT activities remained within the normal range for birds [5, 32, 33]. This finding implies that, despite alterations in certain enzymatic activities, overall liver function appears to be unimpeded. When combined with the data from renal function biomarkers, it allows us to conclude that the birds were not subjected to any toxicological hazards in this experimental setup. This data indicates that incorporating *A. platensis* into the diets did not detrimentally impact hepatic or renal health, further highlighting its potential as a beneficial dietary supplement for broilers.

The different diets significantly impacted the lipid profile. Our findings demonstrate a uniform pattern of increase in total cholesterol, LDL-cholesterol, HDL-cholesterol, VLDL-cholesterol, triacylglycerols and total lipids when *A. platensis* was administered as the sole supplement. To our knowledge, this increase in lipid levels due to Spirulina supplementation may be attributable to enhanced fat absorption in the intestinal tract [34]. However, what stands out is the ability of both enzyme mixes, VemoZyme^®^ P and porcine pancreatin, to reverse this increase, bringing the lipid levels in line with those observed in the control birds. This effect suggests that these enzyme mixtures could potentially exert a beneficial hypolipidemic effect, supporting the digestion of *A. platensis*, which aligns with their primary function. Therefore, the current study does not corroborate the cholesterol and general lipid-lowering properties of *A. platensis* when given alone, as previously reported for some microalgae species [34]. The mechanisms underlying this hypocholesterolaemic action are yet to be fully elucidated.

Plasma total protein levels were found to be consistent across all experimental diets, even though an increase was anticipated in birds fed *A. platensis* diets. With protein levels ranging from 50 to 70% [24], Spirulina is an abundant source of protein, positioning it as a possible substitute for traditional protein sources such as soybean meal [35–37]. Fluctuations in the levels of the acute phase protein, C-reactive protein, lack physiological significance in this context, as the average values obtained among different dietary treatments were quite similar and accompanied by the minimal standard error.

Typically, common electrolytes like sodium and potassium are linked with renal failure pathologies [38]. The kidney plays a pivotal role in maintaining the body’s chloride balance and transport, which is closely tied to sodium transport [39]. Even though statistically significant, the fluctuations observed in plasma sodium levels, which were increased by *A. platensis* alone and mildly reduced by the combination of porcine pancreatin, are not substantially meaningful in physiological terms. The available research on the impacts of Spirulina as a poultry feed ingredient is relatively limited. Consequently, the discrepancies observed in response to biochemical, hepatic and renal metabolites across different experimental trials could be partially attributed to factors such as the dosage and origin of the *A. platensis*, the length of the experimental period, and specific experimental settings. Additionally, it’s important to note that the primary components of *A. platensis* biomass are known to vary based on strain, geographical location, maturity, harvesting season and cultivation requirements [7, 24, 40].

The liver serves as the primary lipogenic tissue in poultry [41]. In our study, the dietary inclusion of 15% *A. platensis*, with or without both enzyme mixes, positively influenced the hepatic fatty acid composition of broilers by elevating *n-3* fatty acids, consequently reducing the *n* − 6/*n* − 3 PUFA ratio. The surge in *n-3* long-chain PUFA in broilers’ livers fed with *A. platensis*, particularly eicosapentaenoic acid (EPA, 20:5*n-3*), docosapentaenoic acid (DPA, 22:5*n-3*) and docosahexaenoic acid (DHA, 22:6*n-3*), can be ascribed to the *de novo* lipogenesis of the precursor alpha-linolenic acid (ALA, 18:3*n-3*), which is abundant in *A. platensis* diets. Indeed, the inclusion of Spirulina resulted in an approximately twofold increase of alpha-linolenic acid in the liver relative to the control diet. The escalation of hepatic *n-3* long-chain PUFA results from the heightened expression and activity of key enzymes like fatty acid desaturases and elongase enzymes, which are engaged in PUFA synthesis [42]. The observed *n-3* PUFA enrichment in the liver can be attributed to the downregulation of PUFA oxidation-related genes, attenuation of the lipid peroxidation cascade, and enhancement of antioxidant characteristics [43]. Contrary to our findings, Madeira et al. [21] found that high dietary *A. platensis* incorporation had a marginal impact on the hepatic fatty acid composition and transcriptional profile of lipid-sensitive mediators in post-weaned piglets. Moreover, we noted that the inclusion of 15% *A. platensis*, alone or with both enzymes, in broiler diets facilitated a decrease in hepatic total lipids without affecting total cholesterol concentration.

Our investigation also extended to evaluating the influence of *A. platensis* diets, with or without enzyme addition, on tocopherol and pigment levels in the liver. Numerous bioactive components derived from algae, including antioxidants, pigments, vitamins and polysaccharides, are acknowledged for their health benefits for both animals and humans. Spirulina is a rich vitamin source with potent antioxidant properties that can alleviate inflammation. Vitamin E, a fat-soluble nutrient found in a variety of foods, serves as an antioxidant in the body, shielding cells from free radical damage [44]. Although diet did not significantly affect vitamin E compound levels, α-tocopherol was the dominant vitamin E homologue detected in all experimental groups, which is consistent with dietary composition, while γ-tocopherol along with β-tocotrienol were minor constituents. Conversely, β-carotene (a precursor of vitamin A), chlorophyll-*a* and total chlorophyll, were elevated by *A. platensis*, which aligns with its inherent nutritional profile [2, 45]. This reflects the bioavailability of these dietary constituents. Chlorophylls and carotenoids are natural lipophilic pigments that maintain antioxidant homeostasis [46], playing a significant role in animal and human health [47].

Spirulina is a rich repository of the most crucial minerals [48], positioning it as an apt dietary supplement for animals [49]. Specifically, copper, iodine, iron, potassium and zinc, which play central roles in numerous physiological processes like cellular metabolism (for instance, iodine) or osmotic regulation (such as sodium), are found abundantly in microalgae [48, 50]. Hence, we examined the impact of *A. platensis* diets on the hepatic mineral content of broilers in this study. The total concentration of macrominerals remained consistent across Spirulina diets, but sodium and magnesium displayed divergent changes. Sodium levels increased in birds fed Spirulina diets, which is a positive development. As an essential nutrient, sodium regulates normal cellular homeostasis, fluid and electrolyte balance and blood pressure. It is also vital for muscle and nerve cell excitability and nutrient and substrate transport across plasma membranes [51]. Although magnesium levels decreased in birds fed *A. platensis* diets, the reduction was minimal. Magnesium, a multifunctional element in the human body, acts as a cofactor for over 300 enzymes, influencing key functions like muscle contraction, neuromuscular conduction, glycaemic control, myocardial contraction and blood pressure regulation [52]. In contrast, the inclusion of *A. platensis* and the supplementation with enzymes did not affect micromineral levels. Zinc, manganese and copper, key cofactors of antioxidant enzymes such as superoxide dismutase [53], form the first line of antioxidant defence. Iron, an essential life-supporting metallic element, plays a critical role in generating harmful oxygen species via the Fenton reaction, resulting in the potent hydroxyl radical. Iron is carried, utilized and stored within specific proteins like transferrin, ferritin, and haemoproteins [54, 55]. Overall, the micromineral findings suggest the maintenance of the birds’ redox balance.

This study reveals novel findings of long-term feeding with a high level of *A. platensis*, which was never been tested before. Even so, it is believed that a wide range of dietary levels of *A. platensis* incorporation should be tested in order to understand from which level forwarded will negatively compromise the growth performance of birds. This remains to be elucidated.

## Conclusion

In summary, key findings suggest that a 15% *A. platensis* diet did not change the haematological profile but increased systemic lipemia in broilers. However, these effects were effectively mitigated by the addition of both enzyme mixes, indicating potential hypocholesterolaemic properties of the commercial enzyme mix VemoZyme^®^ P and porcine pancreatin.

Furthermore, *A. platensis* intake reduced total lipids and enhanced hepatic fatty acid composition by elevating the levels of beneficial *n-3* PUFA, significant to both animal and human health. The diterpene profile, on the other hand, remained unaffected by the dietary alterations. Additionally, antioxidant-rich pigments, such as β-carotene and chlorophyll-*a*, were found to be significantly higher in broilers fed *A. platensis*, independent of commercial enzyme mixture supplementation. Except for minor variations in sodium and magnesium, hepatic minerals largely remained stable under these experimental conditions.

Discriminant analysis utilizing the plasma biochemical profile revealed clear separations among the experimental groups, indicating distinct diet-induced impacts on plasma metabolites. Conversely, hepatic chemical characterization failed to distinguish the effect of both enzyme mixtures.

Our findings illustrate the putative role of *A. platensis* as an alternative source of nutrition for poultry. However, future studies are in need to screen and evaluate the viability of higher incorporation levels of *A. platensis* in the long run, particularly in light of the negative impact observed here on broilers’ growth performance. Moreover and looking forward to, it will be crucial to search for distinct enzymatical, mechanical, chemical and physical Spirulina pre-treatments. If successful, this research could pave the way for a broader utilization of Spirulina, and possibly other microalgae species, taking advantage of all their potential in the field of poultry nutrition.

## Methods

### Animals and feeding protocol

The study was conducted in strict adherence to the European Union legislation guidelines (2010/63/EU Directive), and the experimental procedures received approval from the Ethics Commission of CIISA/FMV, the Animal Care Committee of the National Veterinary Authority (Direção Geral de Alimentação e Veterinária, Portugal), and ORBEA/ISA (protocol code number 0421/000/000/2022).

A cohort of one hundred and twenty, one-day-old male Ross 308 broiler chicks purchased to Pinto Valouro (Bombarral, Portugal), each with an average body weight of 43.6 ± 0.35 g, were accommodated in 40 wire-floored cages for a period of 35 days under environmentally controlled conditions. The temperature and ventilation were monitored continuously by the previous methodology [3, 4]. To abide by the 3R’s principle (Reduce, Refine and Replace) and minimize the number of animals involved in the study, each cage housed three birds, with ten replicate cages assigned to each experimental diet.

For the first seven days, the birds were provided with a corn and soybean meal-based starter diet. Subsequently, from day 7 to day 35, they were switched to one of four grower experimental diets offered *ad libitum* each day. The diets comprised: (1) a control diet based on corn-soybean meal (control, *n* = 10); (2) the control diet enriched with 15% of *A. platensis* powder (Allmicroalgae, Pataias, Portugal) (SP, *n* = 10); (3) the SP diet supplemented with 0.025% of VemoZyme^®^ P (VEMO, Sofia, Bulgaria) (SPV, *n* = 10); (4) the SP diet supplemented with 0.10% of porcine pancreatin (Merck, Darmstadt, Germany) (SPP, *n* = 10). VemoZyme^Ⓡ^ P is a commercial peptidase (15000 tyrosine units/g) with additional α-amylase activity (400 amylase activity units/g), whereas porcine pancreatin is an enzyme mixture primarily containing amylase (7500 FIP-U/g), lipase (6000 FIP-U/g) and peptidase (350 FIP-U/g). The feed ingredients and additives of experimental diets are shown in Table 6.

**Table 6:** Ingredients (% as-fed basis) of the growth experimental diets offered to broilers

Ingredients	Control	SP	SPV	SPP
Corn	55.6	62.2	62.2	62.2
Soybean meal	36.9	18.6	18.6	18.6
Sunflower oil	4.00	1.00	1.00	1.00
Sodium chloride	0.300	0.400	0.400	0.400
Calcium carbonate	1.08	1.39	1.39	1.39
Dicalcium phosphate	1.60	0.900	0.900	0.900
DL-Methionine	0.120	0.030	0.030	0.030
L-Lysine	0	0.050	0.050	0.050
Vitamin-mineral premix^1^	0.400	0.400	0.400	0.400
*Arthrospira platensis* powder	0	15.0	15.0	15.0
VemoZyme^®^ P	0	0	0.025	0
Porcine Pancreatin	0	0	0	0.100

Diets: Control (corn/soybean), SP (15% *A. platensis*), SPV (15% *A. platensis* plus 0.025% VemoZyme^®^ P), SPP (15% *A. platensis* plus 0.10% porcine pancreatin)

^1^Premix composed per kilogram of diet: pantothenic acid 10 mg, vitamin D¬3 2400 IU, cyanocobalamin 0.02 mg, folic acid 1 mg, vitamin K3 2 mg, nicotinic acid 25 mg; vitamin B6 2 mg, vitamin A 10,000 UI, vitamin B1 2 mg, vitamin E 30 mg, vitamin B2 4 mg, Cu 8 mg, Fe 50 mg, I 0.7 mg, Mn 60 mg, Se 0.18 mg, Zn 40 mg

### *Arthrospira platensis* and experimental diets analysis

Table 7 showcases the proximal composition of *A. platensis* and the experimental diets. The dry matter, crude protein, crude fat and ash content were ascertained following the AOAC [56] methodologies. The gross energy content in both *A. platensis* and the diets was determined using adiabatic bomb calorimetry (Parr 1261, Parr Instrument Company, Moline, IL).

**Table 7:** Proximal and nutrient composition of microalga and growth experimental diets offered to broilers

Item	Spirulina	Control	SP	SPV	SPP	
***Gross Energy***,*** kcal/kg as dry matter***	4476	4572	4415	4428	4403	
***Proximate composition***,*** % as dry matter***						
Dry matter	94.8	88.6	88.9	88.9	88.9	
Crude protein	52.5	23.6	23.5	23.1	23.3	
Crude fat	6.56	7.88	5.19	5.30	5.14	
Ash	21.6	6.41	8.21	8.27	8.19	
***Fatty acid profile***,*** % total fatty acids***						
14:0	0.432	0.072	0.078	0.119	0.113	
16:0	39.7	9.90	16.1	16.2	15.9	
16:1*c*9	4.95	0.133	0.796	0.797	0.776	
17:0	0.410	0.065	0.105	0.122	0.123	
17:1*c*9	1.88	0.035	0.215	0.222	0.221	
18:0	1.75	3.05	3.19	2.56	2.58	
18:1*c*9	6.34	31.8	27.1	26.7	27.4	
18:2*n-6*	17.8	51.1	45.4	46.3	45.9	
18:3*n-3*	15.8	1.27	3.36	3.43	3.46	
20:0	0.189	0.379	0.415	0.426	0.431	
20:1*c*11	0.911	0.160	0.204	0.206	0.215	
22:0	0.247	0.580	0.383	0.391	0.393	
***Diterpene profile***,*** µg/g***						
α-Tocopherol	−	58.1	57.3	57.1	47.6	
α-Tocotrienol	−	4.37	4.36	3.77	4.29	
β-Tocopherol	−	0.514	0.360	0.367	0.342	
γ-Tocopherol + β-Tocotrienol	−	7.74	6.96	7.46	6.81	
γ-Tocotrienol	−	11.0	10.1	10.6	10.2	
δ-Tocopherol	−	0.904	0.752	0.687	0.751	
***Pigments***,*** µg/g***						
β-Carotene	−	1.34	115	121	107	
Chlorophyll-*a*^1^	−	13.3	504	517	538	
Chlorophyll-*b*^2^	−	26.8	74.0	67.0	70.9	
Total chlorophylls^3^	−	40.0	578	584	608	
Total carotenoids^4^	−	2.17	152	154	161	
Total chlorophylls + Carotenoids^5^	−	42.2	730	738	769	
***Mineral profile***,*** mg/kg dry matter***						
Calcium	12,010	14,202	18,735	22,143	20,110	
Copper	946	13.2	15.3	16.7	18.4	
Iron	129,528	156	241	317	284	
Magnesium	8894	1955	1526	1912	1869	
Manganese	67.5	71.0	79.0	96.0	92.3	
Phosphorous	10.6	7098	5931	6996	6599	
Potassium	3230	11,961	10,587	11,501	11,161	
Sulphur	67.5	2929	3098	3537	3261	
Sodium	45,193	2991	8598	10,314	9456	
Zinc	99.0	89.3	76.8	93.6	89.3	

Diets: Control (corn/soybean), SP (15% *A. platensis*), SPV (15% *A. platensis* plus 0.025% VemoZyme^®^ P), SPP (15% *A. platensis* plus 0.10% porcine pancreatin)

^1^Ca = 11.24 × A_662_ − 2.04 × A_645_

^2^Cb = 0.13 × A_645_ − 4.19 × A_662_

^3^Ca+b = 7.05 × A_662_ + 18.09 × A_645_

^4^Cx+c = (1000 × A_470_ − 1.90 × Ca − 63.14 × Cb) / 214

^5^(Ca + b) + (Cx + c)

Fatty acids in *A. platensis* and the experimental diets were analysed post a one-step extraction and acidic methylation, following the procedure outlined by Sukhija and Palmquist [57]. The fatty acid methyl esters (FAME) were then examined using gas chromatography integrated with a flame ionization detector (HP7890A Hewlett-Packard, Avondale, PA), utilizing a Supelcowax^®^ 10 capillary column (30 m × 0.20 mm i.d., 0.20 μm film thickness; Supelco, Bellefonte, PA, USA), based on the protocol of Alfaia et al. [4]. FAME within the samples were identified through comparison with a standard FAME mixture (37 Component FAME mix C4-C24, Supelco, Bellefonte, PA, USA). The heneicosanoic acid (21:0) methyl ester served as the internal standard for quantifying individual fatty acids, with fatty acids expressed as a percentage of total fatty acids.

The experimental diets’ β-carotene and diterpenes (vitamin E analogues: tocopherols and tocotrienols) were examined following the protocol described by Prates et al. [58]. Each sample (100 mg) was weighed in duplicate, fortified with ascorbic acid (vitamin C), and subjected to a saponification reaction in a water bath at 80 °C for 15 min. Post extraction and centrifugation (2500 rpm, 10 min), the *n*-hexane phases were analysed with an HPLC system (Agilent 1100 Series, Agilent Technologies Inc., Palo Alto, CA, USA) on a normal-phase silica column (Zorbax RX-Sil, 5 μm particle size, 250 mm × 4.6 mm i.d.). β-carotene was identified with a UV–visible photodiode array detector (λ = 450 nm), and tocopherols and tocotrienols with a fluorescence detector (excitation λ = 295 nm and emission λ = 325 nm). Standard curves of peak area *versus* concentration were utilized to quantify β-carotene and vitamin E analogues.

The pigment profile of the experimental diets was determined with slight modifications to the methods described by Teimouri et al. [59] and Pestana et al. [3]. Briefly, 0.1 g of samples were incubated with 3 mL of acetone, stirred overnight in darkness, and then subjected to centrifugation (4000 rpm for 5 min). Chlorophylls a and b were detected at 662 nm and 645 nm, respectively, and total carotenoids at 470 nm using UV-Vis spectrophotometry (Genesys 150 UV/visible spectrophotometer, Thermo Fisher Scientific, Waltham, MA, USA). The pigment content was calculated using Hynstova et al. [40] equations.

The mineral content of *A. platensis* and the experimental diets was determined by weighing 0.3 g of samples into a digestion tube, adding nitric acid (65%) and hydrochloric acid (37%), and incubating in a ventilated chamber for 16 h. This was followed by the addition of hydrogen peroxide (30%), as described by Ribeiro et al. [60]. After the samples were digested at 95 °C, the resulting solution was analysed using Inductively Coupled Plasma – Optical Emission Spectrometry (ICP-OES, iCAP 7200 duo Thermo Scientific, Waltham, MA, USA), with quantification accomplished through standards and calibration curves.

### Slaughtering and sample collection

Birds and feeders were weighed every week to calculate average daily feed intake (ADFI), average daily gain (ADG), and feed conversion ratio (FCR). At the end of the trial, one 35-day-old broiler from each pen was selected for slaughter, using electrical stunning followed by manual exsanguination. Blood samples were collected in Sarstedt tubes (Numbrecht, Germany), and subsequently centrifuged to obtain plasma. Liver samples were collected from chilled carcasses, once the carcass temperature reached 4 °C. These samples were minced, vacuum-sealed, and stored at -20 °C for future analysis. The remaining birds were euthanized as described above.

### Blood cells and plasma metabolites analysis

As delineated by Madeira et al. [21], the counts of red blood cells, white blood cells and thrombocytes were accomplished utilizing Sysmex XN-10 analysers (Sysmex Corporation, Kobe, Japan). The quantification of red blood cells was achieved through the impedance variation method, post-hydrodynamic focusing. The differential counting of white blood cells (%) was performed on blood smears, which were decolourized using the May-Grünwald-Giemsa technique. The concentration of haemoglobin was assessed photometrically (Genesis 150 UV/visible spectrophotometer, Thermo Fisher Scientific, Waltham, MA, USA), employing sodium lauryl sulphate as the reagent.

Key plasma metabolites including glucose, urea, creatinine, total protein and various lipid forms (triacylglycerols - TAG, total cholesterol, HDL-cholesterol and LDL-cholesterol), as well as enzymatic activities of ALT (alanine aminotransferase, EC 2.6.1.2), AST (aspartate aminotransferase, EC 2.6.1.1), ALP (alkaline phosphatase, EC 3.1.3.1) and GGT (gamma-glutamyltransferase, EC 2.3.2.13) were measured utilizing Roche Diagnostics’ Modular Hitachi Analytical System diagnostic kits (Mannheim, Germany). Formulas by Friedewald et al. [61] and Covaci et al. [62] were employed to calculate VLDL cholesterol and total lipids, respectively. C-reactive protein was quantified via immunoturbidimetry (Roche Diagnostics, Meylan, France). Major electrolytes such as Na^+^, K^+^ and Cl^−^ were determined through indirect potentiometry.

### Determination of total lipids and fatty acid composition in the liver

Total lipids were extracted from the freeze-dried liver samples, in duplicate, utilizing a dichloromethane: methanol (2:1 v/v) mixture, and quantified through gravimetric analysis [63]. The fatty acid composition in the liver samples was identified similarly to the methodology for *A. platensis* and the experimental diets. However, a sequential alkaline and acidic transesterification process was used to transform the fatty acids into FAME, as opposed to acidic methylation. The chromatographic conditions adhered to the protocol previously outlined by Alfaia et al. [4].

### Determination of total cholesterol, β-carotene, diterpenes and pigments in the liver

Liver samples (750 mg each) were used in duplicate to extract total cholesterol, β-carotene and diterpenes, employing the same procedure outlined for the experimental diets. This involved direct saponification, single *n*-hexane extraction and HPLC analysis [58].

For the extraction of pigments (chlorophyll-*a*, chlorophyll-*b* and total carotenoids) from liver samples, a slightly modified version of the method used for the experimental diets was followed. Briefly, 1.5 g of the liver was combined with 3 mL of acetone and homogenized in the dark for 1 min. After centrifugation (3000 rpm for 5 min at 4 °C), the supernatant was isolated and analysed following the conditions set out by Coelho et al. [5]. The quantity of pigments was determined using the equations provided by Hynstova et al. [40].

The hepatic mineral profile was determined following the procedure used for *A. platensis* and diets, except for bromide, which was determined by the method outlined by Delgado et al. [64].

### Statistical analysis

Data were analysed using the Generalized Linear Mixed (GLM) model in the Statistical Analysis System (SAS) program (SAS Institute Inc., Cary, NC) [65], where the experimental diet was included as the main effect in the statistical model. The Kolmogorov-Smirnov test was applied for confirmation of the normality of the distribution of data. For multiple comparisons of least squares means, the adjusted Tukey-Kramer method (PDIFF option) was applied. The experimental unit varied based on the parameters evaluated: the cage was used for body weight, body weight gain, feed intake and feed conversion ratio, while the bird was used for plasma metabolites and overall hepatic parameters. All values were presented as the mean and SEM (standard error of the mean). Statistical significance was considered for p-values less than 0.05.

To assess relationships between blood parameters and all hepatic variables, a Principal Component Analysis (PCA) was performed using SPSS Statistics for Windows (IBM Corp., 2020 release, version 27.0, Armonk, NY, USA).

## Data Availability

All data produced in this study are included in the published version. Datasets are accessible from the corresponding author on request.

## Abbreviations

ADFI: Average daily feed intake
ADG: Average daily gain
ALT: Alanine aminotransferase
ALP: Alkaline phosphatase
AST: Aspartate aminotransferase
FAME: Fatty acid methyl esters
FCR: Feed conversion ratio
GGT: Gamma-glutamyltransferase
HDL: High-density lipoprotein cholesterol
LDL: Low-density lipoprotein cholesterol
MUFA: Monounsaturated fatty acids
PUFA: Polyunsaturated fatty acids
SFA: Saturated fatty acids
TAG: Triacylglycerols
VLDL: Very-low-density lipoprotein cholesterol
